# Genomic Arrangement of Regulons in Bacterial Genomes

**DOI:** 10.1371/journal.pone.0029496

**Published:** 2012-01-03

**Authors:** Han Zhang, Yanbin Yin, Victor Olman, Ying Xu

**Affiliations:** 1 Computational Systems Biology Laboratory, Department of Biochemistry and Molecular Biology and Institute of Bioinformatics, University of Georgia, Athens, Georgia, United States of America; 2 Department of Automation and Intelligent Science, College of Information Technical Science, Nankai University, Tianjin, China; 3 BioEnergy Science Center, United States of America; 4 College of Computer Science and Technology, Jilin University, Changchun, Jilin, China; J. Craig Venter Institute, United States of America

## Abstract

Regulons, as groups of transcriptionally co-regulated operons, are the basic units of cellular response systems in bacterial cells. While the concept has been long and widely used in bacterial studies since it was first proposed in 1964, very little is known about how its component operons are arranged in a bacterial genome. We present a computational study to elucidate of the organizational principles of regulons in a bacterial genome, based on the experimentally validated regulons of *E. coli* and *B. subtilis*. Our results indicate that (1) genomic locations of transcriptional factors (TFs) are under stronger evolutionary constraints than those of the operons they regulate so changing a TF's genomic location will have larger impact to the bacterium than changing the genomic position of any of its target operons; (2) operons of regulons are generally not uniformly distributed in the genome but tend to form a few closely located clusters, which generally consist of genes working in the same metabolic pathways; and (3) the global arrangement of the component operons of all the regulons in a genome tends to minimize a simple scoring function, indicating that the global arrangement of regulons follows simple organizational principles.

## Introduction

Regulons are the basic units of cellular response systems in bacterial cells, and represent a most basic concept in bacterial studies. A bacterial *regulon* is a group of operons that are transcriptionally co-regulated by the same regulatory machinery, consisting of *trans* regulators (transcription factors or simply TFs) and *cis* regulatory binding elements in the promoters of the operons they regulate. Operationally, a regulon contains operons regulated by one same transcription factor. Since the term *regulon* was first proposed in 1964 [1], 173 regulons have been fully or partially identified in *E. coli* K12 [2] and numerous more in other bacteria e.g. *B. subtilis*. Loosely speaking, regulons can be categorized into two classes: local and global regulons, with the former corresponding to regulons consisting of only a few component operons and the latter having a relatively large number of operons [3]. While the functionalities of the known regulons have been well studied, very little is known about how regulons are organized in a bacterial genome. The only published related work is by Janga *et al.* [4], which found that for small regulons, TFs tend to be located close to their targets (TGs).

We present a computational study here to elucidate the organizational principles of regulons in a bacterial genome. We have carried out our study on *E. coli* K12 and also on *B. subtilis* str. 168 to demonstrate the generality of the results. Our key findings are (1) operons of each regulon tend to form a few closely located clusters along with genome; (2) TFs are under stronger evolutionary constraints than their TGs; and (3) the global arrangement of the component operons of all the (known) regulons in a genome tend to minimize a simple scoring function.

## Results and Discussion

We have examined all the 3,684 regulatory relationships between TFs and their TGs in RegulonDB [2], involving 173 TFs and 729 TGs forming 173 regulons. We assigned genes to operons based on the operon information in the DOOR database (14) (http://csbl1.bmb.uga.edu/OperonDB) of *E. coli* K12. Among these regulatory relationships, 105 TFs are self-regulated; 123 (71%) of the 173 TFs regulate more than one TG; 411 (56%) of the 729 TGs are regulated by more than one TF; and 131 (18%) TGs are also TFs so they are regulated by upper-stream TFs while regulating downstream targets in the global transcription regulation network.

### Operons in a regulon tend to form clusters in terms of their genomic locations

**Figure 1:**
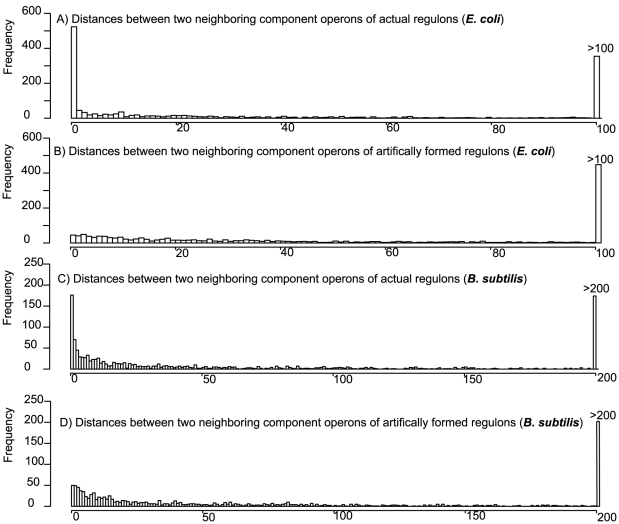
Component operons of regulons tend to be clustered. The distance between two neighboring operons within a regulon is defined as the number of operons between the two operons. The bin width of the histogram is 1. (C) and (D) are similar to (A) and (B), respectively, but are for B. subtilis.

Intuitively we would expect that operons in a regulon should stay close to each other in a genome to facilitate efficient co-regulation, which was used earlier to explain the formation of operons [5]. To test if this is indeed the case, we examined the distribution of the distances between two neighboring operons within a regulon, measured as the (smallest) number of operons between the two operons (we do not consider the orientations of operons). We noted that 523 (32%) of the 1,624 such distances in the 173 regulons are less than two (and 47% less than 10), as shown in Figure 1A, suggesting that the component operons in a regulons tend to cluster together, although they may form multiple clusters. This remains to be true for all large regulons, which are defined as regulons with more than 5 component operons in this study. For example, *crp*, the largest regulon in *E. coli*, consists of 230 operons, 86 of which (37%) have distances less than two. As a control, we have checked a similar distance distribution calculated over 173 artificial regulons by randomly grouping *E. coli* K12 operons. Figure 1B shows the distance distribution, which is clearly very different from the one in Figure 1A. Similar observations were made when studying the regulons of *B. subtilis* (Figure 1C and 1D).

We hypothesize that operons in each cluster of each regulon encode enzymes participating in the same metabolic pathway. To check for this, we consider a (maximal) list of operons of a regulon forms an *operon cluster* if the maximum distance between each pair of neighboring operons in this list is less than five. Using this definition, we obtained 353 operon clusters (85% from large regulons with size >5 as defined above), 242 of which each has at least two operons mapped to some SEED metabolic pathways (http://www.theseed.org/) [6]. Among them, 191 (79%) clusters have at least two operons participating in the same SEED pathway. Interestingly among these 191 clusters, 158 (83%) have all their mapped operons participating in the same SEED pathways. We noted that all these results do not change substantially when we adjust the distance cutoff from 5 to any integer between 2 and 7 when defining an operon cluster (Table S1). This suggests that each operon cluster generally consists of genes working in the same metabolic pathways. Similar observation (Table S2) was made when studying the *B. subtilis* regulons.

### Genomic locations of TFs are under stronger constraints than those of TGs

**Figure 2:**
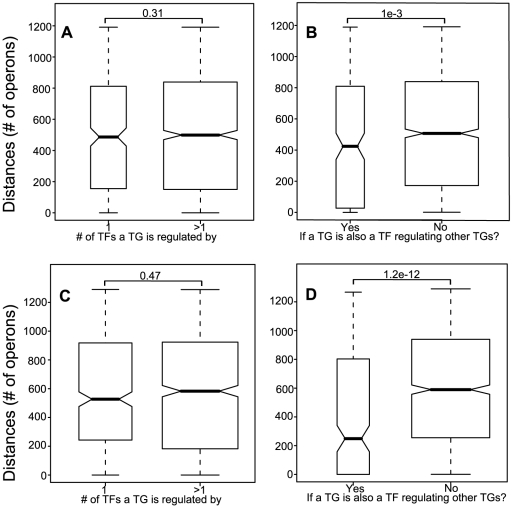
TFs are under stronger constraints than TGs. (A) All TGs are categorized into two groups, TGs regulated by one TF and TGs regulated by multiple TFs. The distributions of the distances (y axis) from TGs to their TFs are shown as box-plots. (B) All TGs are categorized into two groups, TGs that regulate other TGs, and TGs that do not regulate other TGs. The distributions of the distances (y axis) from TGs to their upstream TFs are shown as box-plots. Throughout this paper, the distance between a TF-TG pair is defined as the number of operons between the two operons. (C) and (D) are similar to (A) and (B), respectively, but are for B. subtilis. P-values of Wilcoxon tests are shown between two neighboring boxes.

It has been observed that small regulons tend to have their TFs located close to their TGs [4], suggesting that the genomic location of a TF is under strong constraints from its TGs (meaning TG's locations). A natural question is if a TG is under strong constraints from its TFs. We note that 56% TGs are regulated by more than one TF in *E. coli K12*. From Figure 2A, we conclude that there is no significant difference (Wilcoxon test *P*-value = 0.31) between TGs regulated by one regulator and those regulated by multiple regulators in terms of the distances to their TFs, meaning that an operon does not have to stay close to its regulator even if it is only controlled by one regulator. This is not surprising because the regulator may control many targets, some of which might be close to the regulator while others may not. This finding is opposed to what we found for TFs controlling a small number of operons (Figure S1), suggesting that the genomic locations of *TGs are generally under less constraints than those of TFs*.

To further study this, we divided all TGs into two groups, TGs that are also TFs regulating downstream TGs and TGs that are not TFs. Figure 2B shows that the first group of TGs tends to locate significantly closer (Wilcoxon test *P*-value = 1e−3) to their regulators than the second group, directly suggesting that TGs are under stronger constraints than ordinary TGs from their upstream regulators if they are TFs themselves controlling further downstream targets. This is possibly due to the need for such TGs to have faster reaction time to send the regulatory signal down across the regulatory network.

We have also performed all the analyses for *B. subtilis* str. 168 using data from DBTBS [7] database. The results are shown in Figure 2C and 2D and remain as significant as observed in *E. coli*, strongly suggesting that the observations made above are independent of which bacteria we use and hence may apply to all bacterial organisms in general.

### Global genomic arrangement of regulons

All these observations led to our main hypothesis that the genomic locations of the component operons of all the regulons encoded in a genome are determined by some global organizational principle. Specifically we hypothesize that the global genomic arrangement of regulons tends to minimize the following function based on our preliminary study:
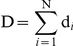
where N is the total number of regulons encoded in a genome and 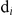 represents the total distance between the genomic location of the TF and all the TGs of the *i^th^* regulon. Note that a similar formula has been used in our recent study on the genomic arrangements of metabolic pathways [8].

**Figure 3:**
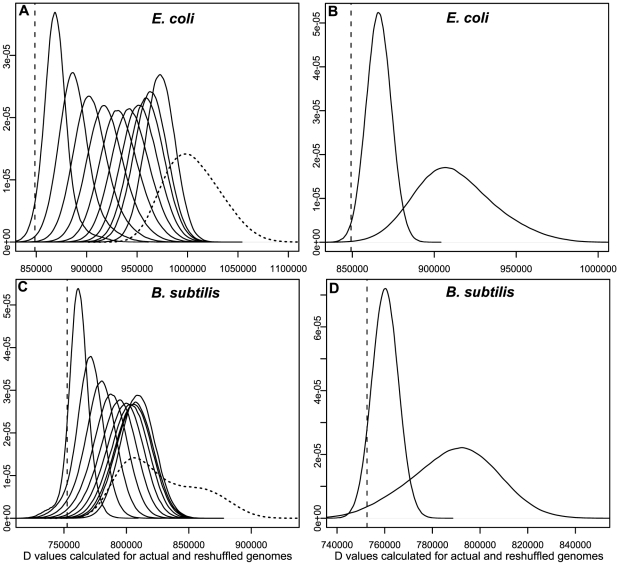
The distributions of D values calculated for the actual and reshuffled genomes. The x-axis represents the D values), and the y-axis is the frequency (density). In (A) each curve is calculated using one million permutations of the current arrangement of the operons in a genome under a specified constraint. Ten D distributions are calculated, with each distribution calculated allowing X% of operons randomly selected among all the operons under consideration and being randomly permutated, with X = 10, 20, …, 100, respectively, where the ten curves from left to right are consistent with the order of X. The vertical dash line shows the D value for the current arrangement of the operons in the E. coli K12 genome. We also conducted permutations using a second manner, i.e. artificially forming regulons and calculating D values for permutated genomes. The result is shown as a dotted curve. (B) A comparison between the D distributions when randomly permuting 100 TGs (curve on the left) versus randomly permuting 100 TFs (curve on the right) in the genome of E. coli K-12. (C) and (D) are similar to (A) and (B), respectively, but are for B. subtilis.

We have used the following procedure to demonstrate that the *D* value of all the known regulons encoded in the *E. coli* genome is significantly smaller than those of the vast majority of alternatively arranged genomes. Specifically, we have considered one million permutations of the genomic locations of X% of operons (both TFs and TGs) of *E. coli* K-12, for X = 10, 20, …, 100 (see **Methods**). Figure 3A shows the *D* value distributions for different percentages of reshuffled locations of operons for the *E. coli* genome. We can clearly see that the current genomic arrangement of operons of *E. coli* K12 has a lower *D* value (the vertical dash line) than the vast majority of the *D* values of the reshuffled genomes, which is also supported by statistical tests (all P-values<0.05, see Table S3). It is also interesting to see that reshuffling TFs increases *D* values considerably more than reshuffling the same amount of TGs (Figure 3B, P-value<0.05), consistent with our observation made based on Figure 2 that TFs are under stronger constraints than TGs in terms of their genomic locations.

For each known regulon in *E. coli* K12, we have also arbitrarily selected the same number of operons from the pool of all operons covered by the known regulons to form an artificial regulon, and do this for every known regulon. Again, we see the *D* value of the real genome (vertical dashed line) is significantly smaller than those of genomes with artificially formed regulons (the dotted curve in Figure 3A).

To ensure that the our observations hold for other bacterial genomes in general, we have checked the observation made in this section on all the 160 known regulons of *B. subtilis*, using the same procedure on *E. coli* genome and the results are as shown in Figure 3C and 3D (see also Table S3), which are clearly highly similar to those shown in Figure 3A and 3B.

This work presents a systematic study of the genomic arrangement of regulons in terms of their organization in a bacterial genome. We made a number of interesting observations related to the organizational principles of regulons in a bacterial genome, namely (1) transcription factors of regulons are under strong constraints from their regulatory targets while TGs do not seem to be under strong constraints from their TFs; (2) regulons tend to form operon clusters, each of which tend to consist of operons encoding the same metabolic pathway; and (3) the genome tends to minimize the overall distance between the TFs and their TGs across all regulons encoded in the genome. We believe that all the observations are mostly due to the need by the cell to efficiently transcribe the relevant genes. Janga et al suggested that TFs of large regulons usually have high expression levels and presumably get to their targets through diffusion, and this might be the reason that they do not need to locate close to their targets. For small regulons, TFs are simply located closely to their targets which should be evolutionarily favored. For larger regulons consisting of multiply clustered operons, the three dimensional packing of the chromosome needs to be considered. It is likely that these organizational principles, along with a few others including genomic organization of metabolic pathways [8], the selfish operon model [9] and the nucleoid compaction [10], [11], [12], [13], [14], collectively determine the local and the global organization of all bacterial genes in a genome [15].

## Materials and Methods

### Date sources

The genome of *E. coli* K-12 MG1655 was downloaded from ftp://ftp.ncbi.nih.gov as of 01/14/2009. All the predicted operons for the organism were downloaded from the DOOR [16] database at http://csbl1.bmb.uga.edu/OperonDB. All regulons data of *E. coli* K-12 MG1655 and of *B. subtilis* str. 168 were downloaded from the RegulonDB [17] and from the DBTBS [17] database, respectively, as of 03/2010.

### Operon shuffling

For each reshuffled genome, the D value defined in the formula was calculated for X = 10, 20, …, 100; X is the percentage of operons to be reshuffled (i.e. their genomic locations are permutated). The following two-step procedure was conducted to randomly shuffle a specified fraction (X%) of operons. We first randomly select operons among all operons of the *E. coli* genome for 10,000 times and then randomly permute their locations 100 times for each specific selection of the 10,000. So we do a total of one million permutations and calculate the D value distribution over the million rearranged genomes

## Supporting Information

Figure S1 **Box plots of the distance distribution of operons (TGs) to their regulators (TFs).** (A) is for E. coli and (B) is for B. subtilis. P-values of Wilcoxon tests are shown between two neighboring boxes.
(EPS)

Table S1 **The number of operon clusters participating in the same SEED pathway under different distance cutoffs (E. coli).** The first column represents the distance cutoff used to define a cluster. The second column is the number of clusters having at least two operons mapped to some SEED metabolic pathways. The third column is the number of clusters having at least two operons participating in the same SEED pathway. The fourth column is the number of clusters having all their mapped operons participating in the same SEED pathways. Regulons with at least two operons are considered.
(DOC)

Table S2 **The number of operon clusters participating in the same SEED pathway under different distance cutoffs (B. subtilis).** See Table S1 legend for details. Note the there are significantly less operons in B. subtilis than in E. coli that are mapped to the SEED pathways. This makes the numbers in Table S2 are much smaller than those in Table S1.
(DOC)

Table S3 **Statistical tests of curves in** **Figure 3** **.** The ‘skewness’ and ‘kurtosis’ columns are calculated to test if the curves in Figure 3 are normal distribution. ‘skewness’ closer to 0 and ‘kurtosis’ closer to 3 indicates close to normal distribution. The ‘P-value’ column is calculated to test if the curves are significantly larger than the vertical dash line, indicating that the permutated genomes have significant larger D values than the actual genomes.
(DOC)
