# Supplementary material for: Genomic Arrangement of Regulons in Bacterial Genomes
Source: PLoS One. 2012 Jan 3;7(1):e29496. doi: 10.1371/journal.pone.0029496 (PMC3250446; doi:10.1371/journal.pone.0029496)
Supplement: Table S1 — The number of operon clusters participating in the same SEED pathway under different distance cutoffs (E. coli). The first column represents the distance cutoff used to define a cluster. The second column is the number of clusters having at least two operons mapped to some SEED metabolic pathways. The third column is the number of clusters having at least two operons participating in the same SEED pathway. The fourth column is the number of clusters having all their mapped operons participating in the same SEED pathways. Regulons with at least two operons are considered. (DOC) [file pone.0029496.s002.doc]

**Table S1**: The number of operon clusters participating in the same SEED pathway under different distance cutoffs (*E. coli*)

| Number  Cutoff | Mapped clusters | At least two operons participating in one pathway | All operons participating in the same pathways |
| --- | --- | --- | --- |
| 2 | 229 | 190 | 178 |
| 3 | 232 | 189 | 165 |
| 4 | 239 | 190 | 161 |
| 5 | 242 | 191 | 158 |
| 6 | 248 | 192 | 155 |
| **7** | 251 | 193 | 152 |
