# Supplementary material for: Genomic Arrangement of Regulons in Bacterial Genomes
Source: PLoS One. 2012 Jan 3;7(1):e29496. doi: 10.1371/journal.pone.0029496 (PMC3250446; doi:10.1371/journal.pone.0029496)
Supplement: Table S2 — The number of operon clusters participating in the same SEED pathway under different distance cutoffs (B. subtilis). See Table S1 legend for details. Note the there are significantly less operons in B. subtilis than in E. coli that are mapped to the SEED pathways. This makes the numbers in Table S2 are much smaller than those in Table S1. (DOC) [file pone.0029496.s003.doc]

**Table S2**: The number of operon clusters participating in the same SEED pathway under different distance cutoffs (*B. subtilis*)

| Number  Cutoff | Mapped clusters | At least two operons participating in one pathway | All operons participating in the same pathways |
| --- | --- | --- | --- |
| 2 | 42 | 28 | 22 |
| 3 | 69 | 38 | 30 |
| 4 | 77 | 40 | 26 |
| 5 | 83 | 50 | 24 |
| 6 | 87 | 44 | 25 |
| **7** | 92 | 44 | 26 |
