# Supplementary material for: Genomic Arrangement of Regulons in Bacterial Genomes
Source: PLoS One. 2012 Jan 3;7(1):e29496. doi: 10.1371/journal.pone.0029496 (PMC3250446; doi:10.1371/journal.pone.0029496)
Supplement: Table S3 — Statistical tests of curves in Figure 3 . The ‘skewness’ and ‘kurtosis’ columns are calculated to test if the curves in Figure 3 are normal distribution. ‘skewness’ closer to 0 and ‘kurtosis’ closer to 3 indicates close to normal distribution. The ‘P-value’ column is calculated to test if the curves are significantly larger than the vertical dash line, indicating that the permutated genomes have significant larger D values than the actual genomes. (DOC) [file pone.0029496.s004.doc]

**Table S3:** Statistical tests of curves in Figure 3

| curve in Fig. 3A | skewness | kurtosis | P-value | curve in Fig. 3B | skewness | kurtosis | P-value |
| --- | --- | --- | --- | --- | --- | --- | --- |
| 10% | 0.754 | 5.222 | 0.04 | 10% | -0.446 | 4.761 | 0.12 |
| 20% | 0.524 | 3.978 | 5E-3 | 20% | -0.278 | 3.656 | 0.06 |
| 30% | 0.399 | 3.490 | 4E-4 | 30% | -0.222 | 3.358 | 0.03 |
| 40% | 0.287 | 3.216 | 3E-5 | 40% | -0.186 | 3.170 | 0.01 |
| 50% | 0.205 | 3.066 | 2E-6 | 50% | -0.167 | 3.069 | 4.7E-3 |
| 60% | 0.147 | 3.005 | 0 | 60% | -0.136 | 3.006 | 1.6E-3 |
| 70% | 0.091 | 2.982 | 0 | 70% | -0.104 | 2.961 | 5.1E-4 |
| 80% | 0.040 | 2.989 | 0 | 80% | -0.080 | 2.937 | 2.1E-4 |
| 90% | 0.002 | 3.016 | 0 | 90% | -0.043 | 2.931 | 9.3E-5 |
| 100% | -0.043 | 3.021 | 0 | 100% | 0.013 | 2.957 | 5E-6 |
|  |  |  |  |  |  |  |  |
| curve in Fig. 3B | skewness | kurtosis | P-value | curve in Fig. 3D | skewness | kurtosis | P-value |
| TG | 0.006 | 3.019 | 0.01 | TG | 0.013 | 3.012 | 0.08 |
| TF | 0.343 | 2.983 | 9.67E-4 | TF | -0.165 | 2.843 | 0.02 |
